# Profiling of Ecstasy Tablets Seized in Iran

**Published:** 2011

**Authors:** Ali Reza Khajeamiri, Farzad Kobarfard, Reza Ahmadkhaniha, Gelareh Mostashari

**Affiliations:** a *Department of Toxicology, School of Pharmacy, Shahid Beheshti University MS, Tehran, Iran.*; b *Department of Medicinal Chemistry, School of Pharmacy, Shahid Beheshti University MS, Tehran, Iran.*; c *Pharmaceutical Sciences Research Center, Tehran University of Medical Sciences, Tehran, Iran.*; d *United Nations Office on Drugs and Crime (UNODC), Tehran, Iran.*; e *Phytochemistry Research Center, Shahid Beheshti University of Medical Sciences, Tehran, Iran.*

**Keywords:** Ecstasy, TLC, Amphetamine-Type Stimulants (ATS), 3,4-methylenedioxymethamphetamine - (MDMA)

## Abstract

In this study 50 samples of ecstasy tablets seized in Iran during the period of 2007 through 2008 were examined and their physical characteristics (appearance, marking, scored/not scored, color, weight, diameter, thickness) were determined. In order to determine the chemical characteristics of these tablets, color tests (Marquis test, Simon’s test, Chen’s test and Gallic acid test), Thin Layer Chromatography (TLC), anion test, residual solvents, Gas Chromatography-Mass Spectrometry (GC-MS) and Liquid Chromatography-Mass Spectrometry (LC-MS) were carried out on the tablets. The range of tablets weight was 96–308 mg and the range of 3,4-methylenedioxymethamphetamine (MDMA) hydrochloride content in these tablets was 60–180 mg. No good correlation was found between the tablets weight and their MDMA contents. All of the tablets containing MDMA had this compound in hydrochloride form. Ketamine, phenmetrazine and ephedrine (or pseudoephedrine) were found in some of the tablets along with MDMA. No MDMA was found in 20% of the tablets. Some of these tablets contained compounds such as caffeine or tramadol as their active ingredient.

## Introduction

Ecstasy is the popular or street name for a substance identified chemically as 3,4-methylenedioxymethamphetamine (MDMA) (1).

MDMA is a ring-substituted amphetamine analog commonly taken as a recreational drug of abuse. It was first synthesized in 1912 by Merck pharmaceuticals and patented in 1914 (2).

Tablets sold as ecstasy primarily contain MDMA, although in some cases other Amphetamine Type Stimulants (ATS) such as methylenedioxyamphetamine (MDA), methylenedioxyethamphetamine (MDEA) and amphetamine are present (3).

There has been a growing tendency among Iranian youth for the abuse of ecstasy tablets during the past decade. ATS active ingredients and tablets are produced in clandestine laboratories without any supervision and quality control over their production which results in impure and bad quality products which may be dangerous in many cases. As a consequence, illicitly manufactured drugs often contain by-products and intermediates stemming from impure starting materials, incomplete reaction and inadequate purification of the final synthetic products (4).

The presence or absence of specific impurities can be useful in determining the synthetic route employed and the starting materials used for the production of ecstasy tablets (5). Chemical profiling has been widely employed as a tool for intelligence purposes such as establishing geographic origins, synthetic routes and distribution routes (6).

One common method of gathering useful information on the source of illicitly–made tablets is by comparison of their physical appearances, *e.g*., markings, colors, sizes and shapes. Nevertheless, tablets having the same physical characteristics are not necessarily associated with the same chemical compositions, since tablets with different chemical compositions could be manufactured with the same dye and have the same markings, sizes and shapes. Therefore, in addition to physical characterization of ecstasy tablets, profiling of chemical compositions with respect to active ingredients, other drugs and the impurities present could yield valuable information for drug intelligence (7).

In the present study 50 samples of ecstasy tablets seized in Iran were provided by antinarcotics police and their physical and chemical characteristics were determined.

## Experimental

d, l-MDMA hydrochloride, d, l-MDA hydrochloride, d, l-MDMA-D5 hydrochloride, d, l-amphetamine hydrochloride, d, l-methamphetamine hydrochloride, ephedrine hydrochloride, pseudoephedrine hydrochloride were purchased from Lipomed pharmaceutical (Switzerland). 

50 different ecstasy tablets seized in Iran during the period of 2007 through 2008 were provided by Antinarcotics police. 

All chemicals were purchased from Merck (Darmstadt, Germany). All solvents were of HPLC grade. 

*Physical characteristics*

Physical characteristics of each tablet including shape, imprint description, break line, color, weight, diameter and thickness were determined. One tablet of each variant was photographed from the front, back and side.

*Preliminary tests*

Preparation of reagents, color tests (Marquis, Simon’s, Chen’s and Gallic acid test) and anion tests (chloride, sulfate and phosphate test) were carried out according to the recommended procedures in UNODC manual (1). For color test in brief 1-2 mg of the powdered tablet was placed in a depression on a spot plate. The proper reagent was then added and the developed color was observed and recorded. 

For each anion test a separate solution was used which had been obtained by dissolving 10 mg of the powdered tablets in 1 mL of distilled deionized water followed by filtration. To the filtrate was added either a few drops of 1.7% silver nitrate solution (for chloride test), 5% barium chloride (for sulfate test) and a mixture of 0.5 mL 10% nitric acid or 0.5 mL 10% ammonium molybdate (for phosphate test). 

*Thin layer chromatography (TLC)*

Stationary phase was silica gel 60 F with the layer thickness of 0.2 mm which contains a fluorescence indicator for visualization under 254 nm UV light.

Solvent systems included: system A (98.5 parts methanol and 1.5 parts concentrated ammonia), system B (85 parts ethyl acetate, 10 parts methanol and 5 parts concentrated ammonia) and C (75 parts cyclohexane, 15 parts toluene and 10 parts diethylamine) (1).

After grinding the tablets, 5 mg of the powder was dissolved in 1 mL methanol and 5 μL of the solution was placed on the TLC plate. 2 μL of mixed standard solution (MDMA, MDA, amphetamine, methamphetamine) and 2 μL of MDMA standard solution (as the most anticipated ATS in the tablets) were placed on the TLC plate. 

*Quantitative analysis*

LC-MS quantitation of MDMA was performed based on a 6-point calibration curve established using reference standards of MDMA HCl over the concentration range of 0.1-4 mg/mL of water. External standard method was used to construct the calibration curve. 

*Chromatographic conditions*

Agilent C18 Zorbax column (3.5 μm, 50 mm by 3 mm) was used as stationary phase.

Mobile phase was composed of acetonitrile (20%), water (80%) and 40 μL of 5 M ammonium formate. pH was adjusted to 2.6 using formic acid. Flow rate was 0.35 mL/min. 

ESI-MS analyses were performed on an Agilent 1100 series ion trap MSD VL instrument (USA) with electrospray ionization source. The temperature of the drying gas was 350 °C and the flow rate was 12 L/min. Nitrogen was used as drying and nebulizing gas. The electrospray voltage was set at 4 KV for the capillary and -400 V for the end plate. Mass detector was adjusted for isolation and selected monitoring of 194 as the hydrogen adduct ion for MDMA. 

*Sample preparation for LC-MS*

Tablets were carefully weighed prior to their grinding. Samples were prepared by dissolving 10 mg of the powdered tablets in 2 mL distilled water followed by filtration through Olimpeak® polypropylene syringe filters (0.45 μm × 25 mm, Teknokroma). 

The concentration of MDMA (mg/mL) in the prepared solution was calculated using the calibration equation. 

The amount of MDMA in each tablet was calculated using the following equation: 

Amount of MDMA in a tablet = (a × w) / 5

In which a is the concentration of MDMA (mg/mL) in the solution prepared by dissolving 10 mg of powdered tablet in 2 mL water. w is weight of the tablet in mg. 

*Qualitative analysis*

GC-MS was used for determination of chemical profile of the ATS tablets. Identification was accomplished by comparing the retention time and mass spectrum of the analyte with that of the reference library. Commercially available Wiley and NIST libraries were used. In order to avoid missing any impurity, two chromatographic system were used:

a) Agilent GC-MS (5973 MSD and 6890 GC system, USA) equipped with HP-5 capillary column.

b) Varian GC-MS (1200L ms and CP-3800 GC, USA) equipped with Factor 4 column (equivalent to HP-1).

*Chromatographic conditions*

Hp-5 or HP-1 (30 m, i.d. 0.25 mm, film thickness 0.25 μm) was used as stationary phase. Carrier gas was helium at flow rate of 1.2 mL/min. Splitless Injection system was used and oven temperature was started at 60 °C, held for 0.5 min and increased to 280 °C at 12 °C/min and held for 30 min. EI was used as ionization mode at 70 eV and mass range was 35-450 amu. 

Sample preparation for *GC-MS*: 5 mg of the powdered tablets was dissolved in 2 mL distilled water. 150 μL of this solution was mixed with 100 μL of d5-MDMA solution (1 mg/mL water). To this solution was added 750 μL of 10% sodium carbonate solution followed by addition of 2 mL pre-distilled diethylether. The solution was mixed 10 min and centrifuged at 3500 rpm. The ether layer was then separated, dried over anhydrous sodium sulfate and evaporated after addition of 2 drops of isopropanol under a gentle stream of nitrogen at 35°C. The residue was reconstituted in 500 μL methanol and 1 μL of this solution was injected into GC.

The aqueous layer was acidified by adding HCl solution (5%) to change pH to 2 followed by extracting with 2 mL diethylether. The ether layer was then separated, dried over anhydrous sodium sulfate and evaporated until totally dried. The residue was reconstituted in 500 μL methanol and 1 μL of this solution was injected into GC.

*Residual solvents*

One tablet and 0.5 g anhydrous sodium sulfate were placed in a vial containing 2 mL distilled water. A septum containing screw cap was used to close the vial and the vial was vortex mixed for 5 min. The vial was then placed in a 70 °C oven for 30 min, after which, one mL of the head space of the vial was drawn into a gas–tight syringe and immediately injected into GC.

*Chromatographic conditions*

Cyanopropyl column (Reastek, 30 m, i.d. 0.25 mm, film thickness 0.25 μm) was used as stationary phase. Carrier gas was helium at flow rate of 1 mL/min.

Oven temperature started at 60 °C, held for 3 min, increased to 250 °C at 10 °C/min ramp and held for 10 min. Injector temperature was adjusted to 150 °C. Mass detector was adjusted on full scan over the mass range of 29-250 amu. The identification of the peaks obtained was carried out by comparison with a commercially available NIST library followed by injecting the head space of a standard sample solution made of identified solvent and sodium sulfate in 2 mL water which had been placed in a 70 °C oven for 30 min.

## Results and Discussion

Full-colour photographs of all tablets, their diameters, thicknesses, weights, amount of MDMA hydrochloride, other active ingredients and/or impurities which were found in some tablets and residual solvents, are presented in Table 1.

**Table 1:** Photographs, selected physical and chemical characteristics of ecstasy tablets seized in Iran during the period of 2007 through 2008

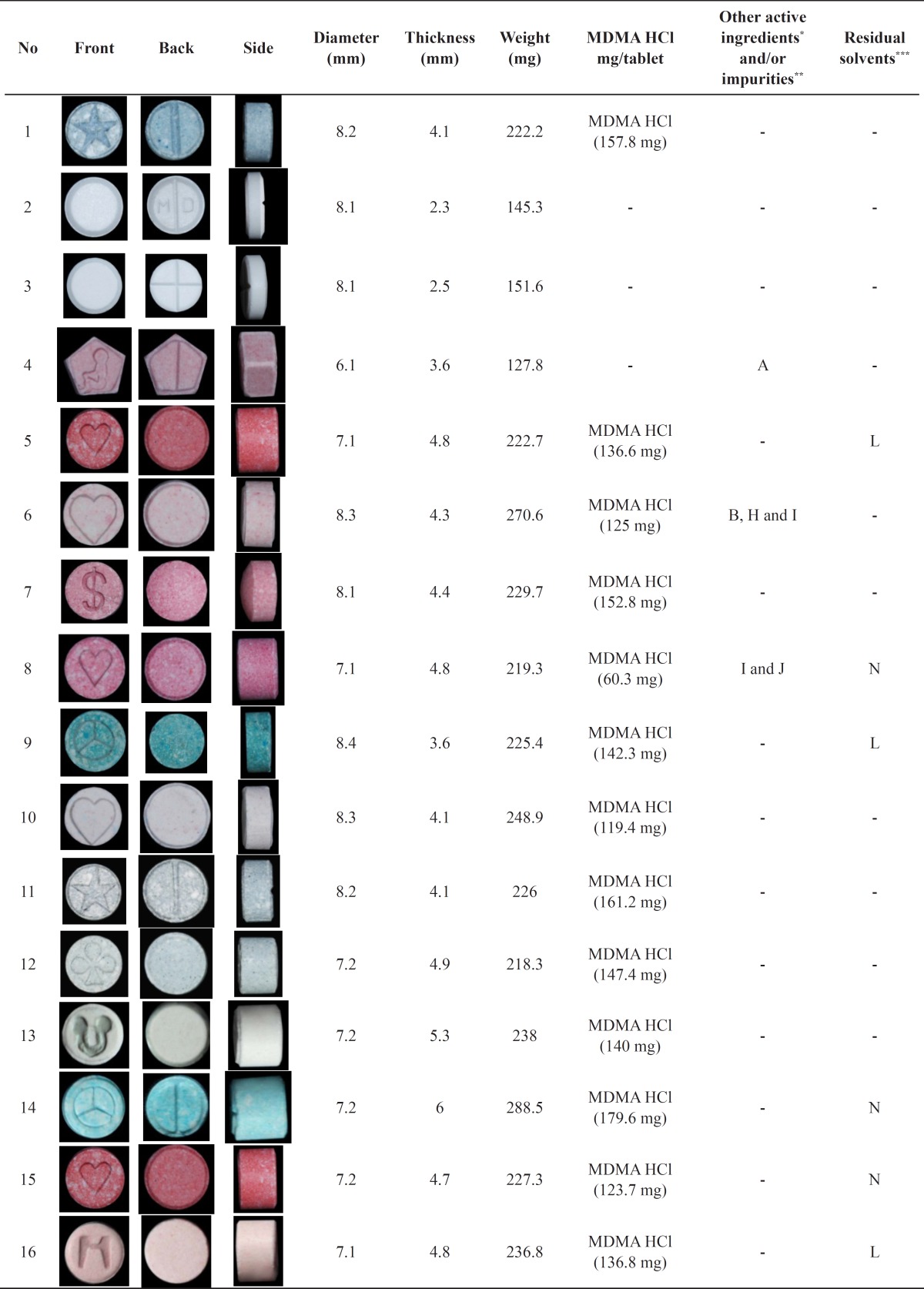

* Active ingredients: A: Caffeine, B: Ephedrine (or pseudoephedrine), C:Ketamine, D: Phenmetrazine, E: Methamphetamine, F: Tramadol.

** Impurities: G: Salsoline, H: 3, 4-methylenedioxyphenylaceton, I: 3, 4-methylenedioxybenzylmethylketoxime, J: N-formyl MDMA, K: *m-*tert-butylphenol. *** Residual solvents: L: Dimethylformamide, M: Methanol, N: Butanol.

The most important color tests for these substances are Marquis, Simon’s, Chen’s and Gallic acid. All tablets containing MDMA gave a dark purple color to Marquis test and dark blue to Simon’s. No color was developed in reaction with Chen’s reagent. Despite the fact that ephedrine (or pseudoephedrine) was found in GC-MS analysis of tablets 6, 27, 33, 35 and 38, they all gave negative results to Chen’s test. This may be due to the low amount of ephedrine (or pseudoephedrine) in the tablets. Gallic acid test provides a simple means for the distinction of MDMA, MDA and MDEA from amphetamine or methamphetamine, because it reacts specifically with methylenedioxy-substituted aromatic compounds. For all tablets containing MDMA a dark green color was developed in response to the addition of Gallic acid reagent. 

Anion tests were conducted to determine the presence of chloride, sulfate and phosphate. All of the tablets containing MDMA and two tablets (tablets 48 and 49) containing tramadol tested positive for chloride anion.

TLC has become one of the most commonly used techniques for the separation and identification of illicitly manufactured drugs. Solvent systems included system A, B and C. We found that the system C is the most suitable system for ecstasy tablets.

**Figure 1:**
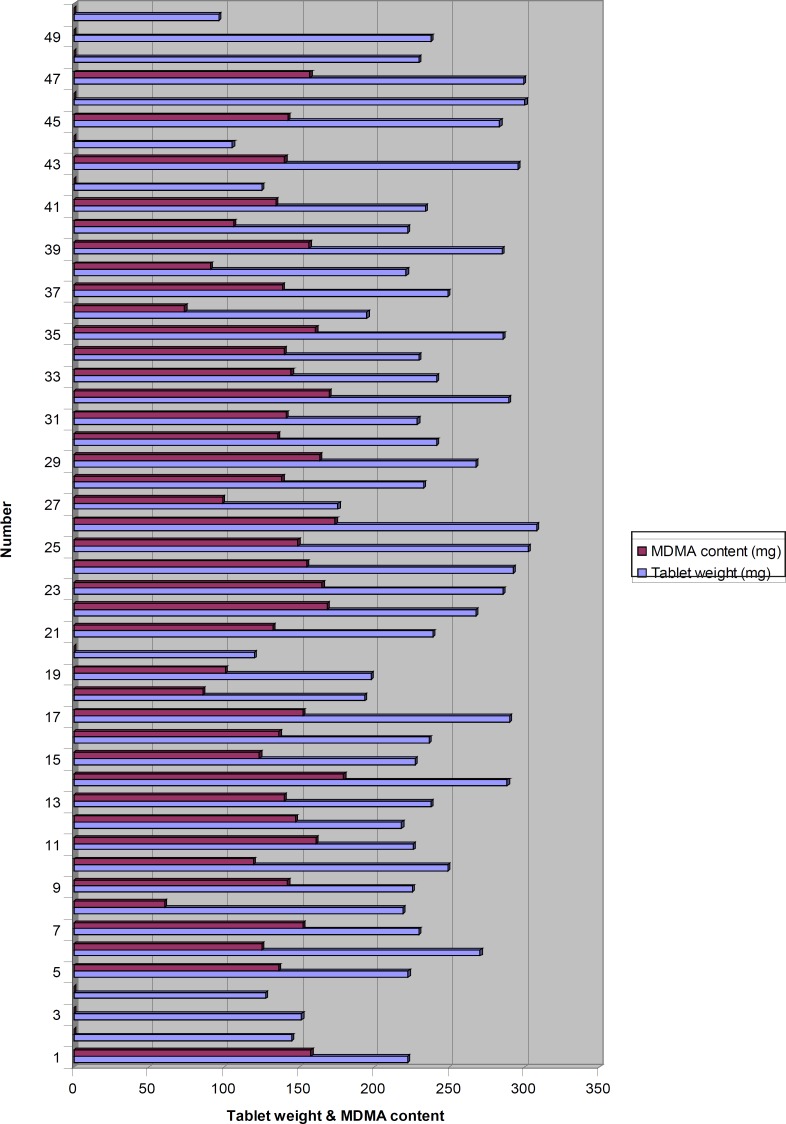
The distribution of tablets weight and MDMA content of the tablets together

In this study, the range of tablets weight was 96–308 mg and the range of MDMA hydrochloride content in these tablets was 60–180 mg. Figure 1 shows the distribution of tablets weight and MDMA content of the tablets together. As it appears in this figure, all the tablets without MDMA (tablets 2, 3, 4, 20, 42, 44, 46, 48, 49 and 50) had much lower weights compared to those with MDMA except for tablets 46, 48 and 49. Tablets 48 and 49 contained tramadol as their active ingredient. Since tramadol tablet is one of the official dosage forms of this drug in Iran, it could be speculated that tablets 48 and 49 were commercially made and are legitimate tablets. All the tablets with unusual thickness (>5 mm, tablets 13, 14, 17, 21, 25, 30, 32, 35, 39, 41 and 45) contained about 150 mg of MDMA hydrochloride. Although it does not mean that the tablets with regular thickness always contain less MDMA. No strong correlation was found between the amount of MDMA and tablets weight. 

**Figure 2:**
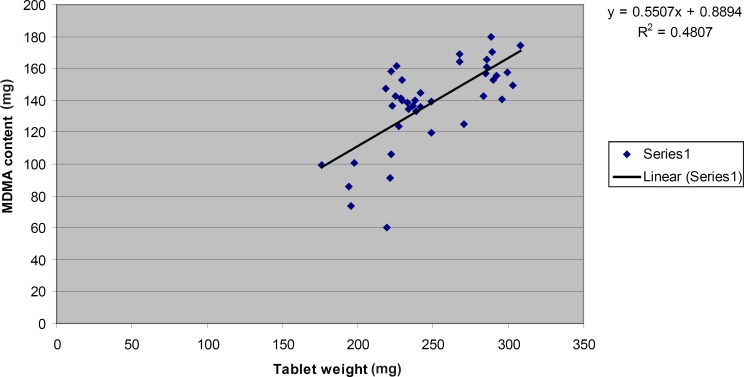
The correlation between “MDMA content” and “tablet weight” for those tablets which contained MDMA as their active ingredient

Figure 2 shows the correlation between “MDMA content” and “tablet weight” for those tablets which contained MDMA as their active ingredient.indeed some rules are needed to be executed in this regard to minimize the contamination to the least possible amount and to preclude its adverse effect. Therefore, due to the importance of baby food and infant formula, more researches are to be conducted for designing quality improvement and safety of food stuff such as milk, infant formula and animal tissues from any drug residues. 

Ten tablets out of 50 contained no MDMA. All the tablets without any specific imprint (tablets 2, 3, 44, 48, 49 and 50) contained no MDMA without any exception. In other words, all the MDMA containing tablets had specific imprints on one of their sides. Meanwhile not all the tablets which had specific imprints contained MDMA (tablets 4, 20, 42 and 46). 

One tablet (tablet 37) contained MDMA and ketamine. One tablet (tablet 38) contained MDMA, ketamine and a compound which was identified as phenmetrazine by library search. This compound appeared at the same retention time as ephedrine (or pseudoephedrine). According to the report by Wille and Lambert a formaldehyde contamination in solvents such as methanol can result in conversion of ephedrine to phenmetrazine in the injection port of GC (8). Since in our method of sample preparation, methanol is used as the final reconstitution solvent, the possibility of the conversion of ephedrine (or pseudoephedrine) to phenmetrazine was investigated by injecting a solution of ephedrine in methanol-formaldehyde (1 : 1, v/v) mixture and a peak was observed at the same retention time as ephedrine which was identified by library search as phenmetrazine. Due to the unavailability of phenmetrazine reference standard, reconfirmation of this finding using the difference in retention time for ephedrine and phenmetrazine was not performed. 

Interestingly tablets 4, 20, 42 and 46 which had no MDMA, had the same imprint. Tablets 4, 20 and 42 were also similar in their diameters, thicknesses, weights and shapes. One tablet (tablet 4) contained caffeine. Some of the tablets contained ephedrine (or pseudoephedrine), salsoline, 3,4-methylendioxybenzylmetylketoxime, 3, 4-methylendioxyphenylacetone, m-tert-butylphenol and *N*-formyl MDMA. 

**Figure 3:**
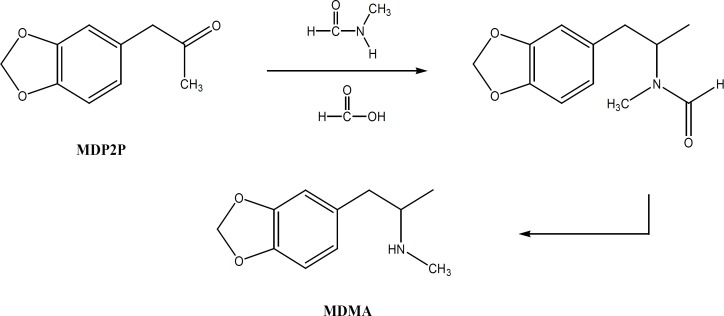
Leuckart reaction for MDMA synthesis

*N*-formyl MDMA is an intermediate in Leuckart method for MDMA synthesis. In this method *N*-formyl MDMA is hydrolyzed with strong acid to produce MDMA (Figure 3). 

It has also been reported that N-formyl MDMA is an impurity in MDMA produced via reductive amination (9). 

The most frequently found imprints among the tablets were heart (tablets 5, 6, 8, 10, 15, 40 and 43), Mercedes (tablets 9, 14, 25, 32, 35 and 39) and mickey mouse (tablets 13, 21, 28, 30 and 41). Although no decisive conclusion could be drawn about the origin of the tablets based on their shapes and imprints in most cases, in certain cases such as the tablets with mickey mouse imprint it appears that they have similar shape, weight and MDMA content and thus they could be considered from the same origin. 

Dimethyl formamide was the most frequent solvent (9 cases) found in the tablets. This could be due to the high boiling point and thus low volatility of this solvent. No plausible correlation was found between the type of residual solvent found in the tablets and their other characteristics. Tablets 24 and 26 had the same appearance and both contained similar amounts of MDMA and yet were different in their residual solvent, suggesting that these tablets may have been made in the same place but they are from different batches. 

*Using d5-MDMA as a tool to verify the efficiency of extraction procedure*: Pentadeutero-methylenedioxymethamphetamine (d5-MDMA) is an isotopically labeled form of MDMA in which five hydrogen atoms are replaced with deuterium and therefore its molecular weight is 5 amu higher than molecular weight of MDMA (198 for d5-MDMA and 193 for MDMA). 

Both MDMA and d5-MDMA will have the same retention time in gas chromatography since they have similar physicochemical properties. However they could be distinguished from each other by mass spectrometery based on their mass difference. d5-MDMA does not naturally exist and therefore the possibility of its presence in the ATS tablets is zero. If d5-MDMA is added to the ATS samples before starting the extraction process, it could serve as a quality control system to verify the efficiency of extraction process. If d5-MDMA peak appears in chromatogram, it could be concluded that the extraction procedure has been efficient and thus if any ATS derivative had existed in the sample they would have been extracted too and if d5-MDMA does not appear in the chromatogram, it would be concluded that somewhere in the extraction process, there has been a mistake or defect and the process of extraction needs to be repeated or modified accordingly. 

In the present study d5-MDMA was added to the sample prior to the start of extraction process mainly for two reasons:

a: To verify the efficiency of the extraction process.

b: To confirm the identity of MDMA peak, based on retention time.

Since d5-MDMA has the same retention time as MDMA, co-elution of d5-MDMA and MDMA will verify the identity of the MDMA peak and no additional spiking and co-injection with standard MDMA solution will be necessary. 
